# The effect of weight loss on HDL subfractions and LCAT activity in two genotypes of APOA-II -265T>C polymorphism

**DOI:** 10.1186/s12937-017-0255-4

**Published:** 2017-05-25

**Authors:** Masoumeh Moradi, Maryam Mahmoudi, Ahmad Saedisomeolia, Roxana Zahirihashemi, Fariba Koohdani

**Affiliations:** 1 Department of Cellular and Molecular Nutrition, School of Nutritional Sciences and Dietetics, International Campus, Tehran University of Medical Sciences, Hojatdoost Ave., Naderi St., Keshavarz Blvd., Tehran, Iran; 2 Department of Cellular and Molecular Nutrition, School of Nutritional Sciences and Dietetics, Tehran University of Medical Sciences, Tehran, Iran; 3 Department of Pharmacology, School of Medicine, Western Sydney University, Campbelltown, NSW Australia; 4 Diabetes Research Center, Endocrinology and Metabolism Clinical Sciences Institute, Tehran University of Medical Sciences, Tehran, Iran

**Keywords:** Apolipoprotein A-II, Polymorphism, HDL subfractions, Obesity, Weight Loss

## Abstract

**Background:**

People may have different responses to the same environmental changes. It has been reported that genome variations may be responsible for these differences. Also, HDL subfractions may be influenced by different genetic variations. The aim of the present study was to determine gene-diet interactions and to evaluate the influence of weight loss on HDL subfractions between two genotypes of -265 T>C APOA-II polymorphism.

**Methods:**

In the present study, 56 overweight and obese patients with type 2 diabetes mellitus were selected from 697 genotype-specified subjects. After matching for gender, age and BMI at the beginning of the study, an equal number of patients remained on each genotype of APOA-II (TT/TC and CC group). After a 6-week calorie restriction program, 44 patients completed the study. Serum HDL subfractions, including HDL2 and HDL3 and LCAT activity, were compared between the two genotypes and, before and after the intervention, were separated in each genotype.

**Results:**

Serum concentration of HDL and its subfractions decreased significantly due to the weight loss. A comparison of the mean changes between the genotypes showed that HDL3 significantly decreased in the CC genotype while, in the TT/TC group, the serum concentration of HDL2 was significantly reduced. However, the increase of LCAT activity was not significant among the two genotypes.

**Conclusion:**

A comparison of mean changes of variables within two genotype groups showed that C homozygote carriers lead to a general shift toward larger size HDL subfractions and T allele carriers shift toward smaller size HDL subfractions after weight loss.

## Background

The growing prevalence of obesity and type 2 diabetes mellitus (T2DM) in the world makes these conditions a global public health problem [1]. Moreover, the incidence of T2DM is linked to obesity, and Asian people develop diabetes at a lower degree of obesity and at a younger age [2]. Both obesity and T2DM mostly result from a changing lifestyle, such as increased daily energy intake and a sedentary lifestyle, yet both are preventable by lifestyle modification [1]. The primary goal for overweight or obese people who have T2DM is to lose weight. A variety of diets have been suggested for treating obesity; however, in general, a calorie restriction diet is recommended for these patients [3]. However, it has reported that people show different responses to similar environmental changes, including dietary modifications. It is suggested that genetic differences may, therefore, be the reason for these variations [4]. One of the candidate genes that is involved here is apolipoprotein A-II (APOA-II). APOA-II is the second most common protein in high-density lipoprotein (HDL) [5].

HDL is secreted from the liver as discoidal particles, which consist of two or more apolipoprotein molecules, phospholipids and unesterified cholesterol [6]. Unesterified cholesterols are substrates for Lecithin: cholesterol acyltransferase (LCAT). The act of LCAT converts discoidal HDL into the large spherical HDL particles [6]. Spherical HDL contain a core of neutral lipids such as triglyceride and cholesteryl esters surrounded by phospholipids, unesterified cholesterol and apolipoproteins, which can be separated by ultracentrifugation into two major subfractions consisting of HDL2 and HDL3 [7]. HDL2 particles are generated when LCAT esterified free cholesterol on the surface of HDL3. Conversely, HDL3 is formed when HDL2 particles are hydrolyzed by hepatic lipase (HL) enzyme [8]. HDL2 particles are larger and less dense than HDL3 [6, 8].

It has been found that APOA-II can modulate the activity of enzymes implicated in HDL metabolism such as LCAT and, consequently, affect the HDL particle size [5]. It has been proven that APOA-I is a potent catalytic activator of LCAT [9]. In vitro studies have shown that APOA-II has more affinity with HDL surface and can displace APOA-I from HDL. This process may impair the function of APOA-I involved in the reverse cholesterol transfer (RCT) [10]. As some studies have shown, in human APOA-II transgenic mice the capacity of HDL to promote cellular cholesterol efflux decreases [11]. In APOA-II transgenic mice, overexpression of APOA-II can reduce LCAT activity through the displacement of APOA-I from the surface of HDL [12].

There are limited studies on the relationship between plasma APOA-II levels and HDL subfractions or LCAT activity in humans. In APOA-II -265 T>C polymorphism, there is a substitution of T-to-C at the 265 position of this gene. This replacement results in a decreased APOA-II expression in the liver and, as a consequence, different serum APOA-II concentrations in two genotypes [13]. It was proposed that -265 T>C APOAII polymorphism is related to anthropometric variables [13, 14], while daily food intake such as saturated fatty acids [15, 16] and similar behaviors have an effect on weight loss [17].

Due to different APOA-II expressions in the liver and, consequently, different serum APOA-II levels in APOA-II -265 T > C polymorphism, we hypothesize that APOA-II genotype might be associated with different levels of LCAT activity and HDL subfractions. Therefore, to test this hypothesis, this study was designed to determine the HDL subfraction changes of two different genotypes after the same lifestyle modification in order to gain a better understanding of the gene-diet interaction.

## Methods

### Study design and diet intervention

In this study, 56 overweight (BMI = 25–29.9 kg/m^2^) and obese (BMI ≥ 30 kg/m^2^) type 2 diabetes (T2D) patients with a mean age of 56.68 ± 5.95 were selected from the 697 genotype-specified subjects in the earlier study [18]. The patients were matched for gender, age, and BMI and, at the baseline of study, an equal number of patients remained on each genotype of APOA-II (28 people in the TT/TC group and 28 people in the CC group). Exclusion criteria were smoking, alcohol consumption, pregnancy, lactation, having certain diseases such as liver and thyroid disease, kidney failure, cancer and use of some medications, including anti-inflammatory agents, nutrient supplements and insulin injection.

Weight, height and waist circumference were measured by a trained dietitian using standard protocols [19]. The total energy requirements were calculated using Mifflin-St. Jeor formula for basal energy expenditure (BEE), then the thermic effect of food (TEF) and activity thermogenesis (AT) were added to the BEE to obtain the total energy expenditure (TEE) for each patient [19]. This dietary program was based on a caloric restriction of 500–1000 kcal/d [3]. In the present study, the diet prescribed provided 750 kcal/d less than their calorific needs. Only two patients in each group received 500 kcal/d less than their daily calorific needs. A food menu was designed with a specified energy and fiber intake (30 gr/d) [20]. The percentage of energy intake was 55–65% of energy from carbohydrates, 20–25% from fats and 15–20% from proteins [21]. The recipes were set on a daily basis for each individual for a period of six weeks by a trained dietitian. Every two weeks, seven menus with the same daily calories were given to the patients. Therefore, patients could choose the desired menu, or even repeat a menu in one specific week. Also, the same amount of calories was calculated for every meal; for example, there were seven lunch menus with the same calorific amounts, enabling the patients to choose their favorite.

The compliance was checked using weekly-based phone calls and, every second week, patients were invited to the clinic in order to measure weight and waist circumference and estimate dietary intakes. A trained dietitian taught the patients how to complete food diaries and, if participants exchanged their food items, they had to write this in their food diaries. Participants who completed ≥ 80% of the planned diets were encouraged to continue for the subsequent weeks, while those subjects who did not complete ≥ 80% of the prescribed diet for one successive week were excluded from the study [22].

Physical activity was evaluated using an international physical activity questionnaire (IPAQ) [23]. The patients were requested to maintain their usual physical activity during the intervention.

### Sample collection and measurement of serum HDL2, HDL3 and LCAT activity

At the baseline of the study and after six weeks intervention, 15–20 ml of fasting blood samples (after 10–12 hours fasting) were taken from the patients and allocated into separate tubes with and without anticoagulant Ethylenediaminetetraacetic acid (EDTA).

The blood samples without EDTA were centrifuged at 3000 rpm for 10 min at 4 °C and serum samples were stored at −80 °C until the analysis. A concentration of serum HDL2 and HDL3 was measured using a commercial ELISA kit (Crystal Day, China; Cat. No: E2188Hu and E2189Hu, respectively) according to the instructions provided by the manufacturer. The serum LCAT activity was measured using a commercial fluorimetric activity assay kit (Roar Biomedical, NY, USA; Cat. No: MAK107) according to the manufacturer’s instructions. LCAT activity was calculated by the changes in 470/390 nm emission intensity. The emission intensities of 390 nm indicate that the substrate hydrolyzed and 470 nm show the substrate is not hydrolyzed.

### Statistical analysis

Statistical analysis was carried out using SPSS 16.0 for Windows 7. The characteristic of the study population is expressed as mean ± standard deviation. The normality of distribution variables was assessed using the Kolmogorov-Smirnov test. The serum HDL2 and HDL3 concentration was log-transformed to normalize the error distribution. The independent t-test and chi-square test were used to compare continuous and categorical variables, respectively, between the two genotypes. A covariance test was used to adjust the confounders of each dependent variable. A paired sample t-test was used to compare the changes within the groups. The APOAII genotypes were analyzed using a comparison of homozygote of CC with the combined homozygote of TT and heterozygote TC (TT + TC). The mean difference was used to distinguish any association between parameters and the genotype groups. The significance level was defined as *P* < 0.05 for all statistical tests.

## Results

At the baseline of the study, 56 patients with T2D were matched by means of gender, age and BMI. In total, 44 patients completed the study (7 men and 15 women in each group), while 12 patients did not complete the study. In the CC genotype group, cancer was diagnosed in a patient during intervention and a patient traveling abroad; in the TT/TC genotype group, a patient caught a severe cold and used corticosteroid drugs, while a patient was dropped to match with the CC group. The other patients were withdrawn because of poor compliance. Finally, 22 patients (7 men and 15 women in each group) completed the study.

The characteristics of the study population based on the genotype groups are shown in Table 1. When the subjects were matched at the baseline of the study, no significant differences were recorded in BMI and waist to hip ratio between the two genotype groups. Also, serum HDL and HDL subfraction levels were not significantly different between the two genotypes at the baseline of the study.

**Table 1:** General characteristic of −265 T>C polymorphism subjects at the baseline

Variable	TT/TC (*n* = 22)	CC (*n* = 22)	*P*_*value*_
BMI(kg/m^2^)			
Overweight	10(45.5)	10(45.5)	0.67^a^
Obese	12(55.5)	12(55.5)	
Waist/Hip	0.96(0.04)	0.97(0.06)	0.72^b^
APOA-II/APOA-I	1.49(0.11)	1.44(0.36)	0.60^b^
HDL(ng/ml)	43.63(8.16)	46.18(7.79)	0.29^b^
HDL2(ng/ml)	16.08(0.3)	14.72(0.29)	0.33^b^
HDL3(ng/ml)	28.04(0.24)	31.99(0.32)	0.13^b^
LCAT activity(470/390)	0.22(0.07)	0.23(0.08)	0.60^b^

Values are presented as mean ± standard deviation for continuous and number (%) for categorical variables

*BMI* Body Mass Index, *APOA-I* Apolipoprotein A-I, *APOA-II* Apolipoprotein A-II, *HDL* High Density Lipoprotein, *LCAT* Lecithin: cholesterol acyltransferase

For HDL2 and HDL3 Geometric Mean Ratio was used

^a^Using the chi-square test ^b^Using independent sample *t*-test^,^

*P*_*value*_ ≤0/05 was considered as statistically significant

After six weeks of calorie restriction, the mean BMI was reduced from 31.07 (±4.39) kg/m^2^ to 29.76 (±4.2) kg/m^2^ and the mean weight was reduced from 81.68 (±13.11) kg to 78.41 (±12.96) kg. The effects of the six weeks intervention on serum HDL and HDL subfractions concentration and LCAT activity in the total population, as well as in the two genotype groups, are summarized in Table 2. In the total population, without considering the genotype, the mean concentration of HDL and HDL2 decreased significantly (*P* < 0.001 and *P* = 0.03, respectively), while LCAT activity increased, but not significantly, after weight loss. However, when compared within the groups, the serum HDL2 level significantly reduced only in the TT/TC group (*P* = 0.005), while within the CC group the reduction of HDL3 was significant (*P* = 0.04). The level of HDL decreased significantly in both groups (*P* = 0.003 in TT/TC group and *P* = 0.04 in CC group). The mean difference of variables between the two genotypes before and after adjusting for physical activity and changes of BMI has been shown in Table 3. There are statistically significant differences in the TT/TC genotype group as compared to the CC genotype group. There were inverse correlations between the mean difference values of serum APOA-II, ratio of APOA-II to APOA-I and HDL3 in the total population and CC genotype. In contrast, these correlations are positive in the TT/TC genotype group (Table 4).

**Table 2:** Outcome variables before and after intervention in all patients and separately between genotypes

Subjects	Variable	Before intervention	After intervention	*P_*value*_
		Mean(SD)	Mean(SD)	
Total (*n* = 44)				
	BMI (kg/m^2^)			
	Normal	-	2(4.5)	
	Overwight	20(45.5)	28(63.5)	≤0.001^a^
	obese	24(54.5)	14(32)	
	Waist/Hip	0.97(0.05)	0.93(0.05)	≤0.001^b^
	APOA-II/APOA-I	1.46(0.26)	1.40(0.17)	0.12^b^
	HDL(ng/ml)	44.90(7.99)	42.76(7.81)	≤0.001*^b^
	HDL2(ng/ml)	15.38(0.29)	14.25(0.28)	0.03*^b^
	HDL3(ng/ml)	29.95(0.29)	28.75(0.28)	0.11^b^
	LCAT activity (470/390)	0.23(0.07)	0.24(0.09)	0.09^b^
TT/TC (*n* = 22)				
	BMI (kg/m^2^)			
	Normal	-	-	
	Overwight	10(45.5)	16(72.7)	0.032^a^
	obese	12(55.5)	6(27.3)	
	Waist/Hip	0.96(0.04)	0.93(0.04)	≤0.001^b^
	APOA-II/APOA-I	1.49(0.11)	1.31(0.16)	0.002*^b^
	HDL(ng/ml)	43.63(8.16)	40.81(6.77)	0.003*^b^
	HDL2(ng/ml)	16.08(0.3)	13.84(0.21)	0.005*^b^
	HDL3(ng/ml)	28.04(0.24)	28.18(0.24)	0.87^b^
	LCAT activity(470/390)	0.22(0.07)	0.24(0.07)	0.35^b^
CC (*n* = 22)				
	BMI (kg/m^2^)			
	Normal	-	2(9)	
	Overwight	10(45.5)	12(54.5)	0.032^a^
	obese	12(55.5)	8(36.5)	
	Waist/Hip	0.97(0.06)	0.93(0.05)	≤0.001^b^
	APOA-II/APOA-I	1.44(0.36)	1.48(0.14)	0.58^b^
	HDL(ng/ml)	46.18(7.79)	44.71(8.44)	0.04*^b^
	HDL2(ng/ml)	14.72(0.29)	14.68(0.34)	0.95^b^
	HDL3(ng/ml)	31.99(0.32)	29.34(0.33)	0.03*^b^
	LCAT activity(470/390)	0.23(0.08)	0.25(0.10)	0.12^b^

Values are presented as mean ± standard deviation for continuous and number (%) for categorical variables

*BMI* Body Mass Index, *APOA-I* Apolipoprotein A-I, *APOA-II* Apolipoprotein A-II, *HDL* High Density Lipoprotein, *LCAT* Lecithin: cholesterol acyltransferase

For HDL2 and HDL3 Geometric Mean Ratio was used

^a^Using the chi-square test, and ^b^ Using paired sample *t*-test

*P_*value*_ ≤0/05 was considered as statistically significant

**Table 3:** Comparison of mean differences of variables between TT/TC and CC genotypes in APOA-II -265 T > C polymorphism

Variable	TT/TC(*n* = 22)	CC(*n* = 22)	95% confidence interval	^a^ *P*_*value*_	^b^ *P*_*value*_
	Mean differences(SD)	Mean differences(SD)			
HDL(ng/ml)	−2.81(3.94)	−1.46(3.14)	−3.5 to 0.82	0.21	0.27
HDL2(ng/ml)	0.86(0.22)	0.99(0.22)	0.75 to 1.008	0.03*	0.05*
HDL3(ng/ml)	1.004(0.14)	0.91(0.17)	0.99 to 1.19	0.06	0.02*
LCAT activity (470/390)	0.24(0.07)	0.25(0.1)	−0.06 to 0.04	0.63	0.52

HDL, High density lipoprotein; LCAT, lecithin: cholesterol acyltransferase

^a^mean differences of variables between two genotype without adjusting for confounders and ^b^after adjusting for differences of BMI and physical activity

*P_*value*_ was obtained from the student’s independent t-test and *P*_*value*_ ≤0/05 was considered as statistically significant

For HDL2 and HDL3 Geometric Mean Ratio was used

**Table 4:** Pearson correlation coefficient between changes of HDL subfraction, LCAT activity and apolipoproteins

Subjects	Variables	HDL	HDL2	HDL3	LCAT activity
		r (*P*_*value*_)	r (*P*_*value*_)	r (*P*_*value*_)	
Total (*n* = 44)	APOA-I	0.25(0.09)	−0.18(0.23)	−0.18(0.23)	−0.02(0.85)
	APOA-II	0.07(0.64)	0.07(0.62)	−0.40(0.007)^a^	−0.04(0.77)
	APOA-II/APOA-I ratio	−0.09(0.55)	−0.03(0.18)	−0.27(0.06)	−0.02(0.88)
TT/TC (*n* = 22)	APOA-I	0.35(0.1)	0.23(0.28)	−0.40(0.05)^a^	−0.006(0.97)
	APOA-II	0.23(0.30)	0.001(0.99)	0.01(0.96)	−0.22(0.31)
	APOA-II/APOA-I ratio	−0.15(0.50)	−0.27(0.22)	0.43(0.04)^a^	−0.21(0.32)
CC (*N* = 22)	APOA-I	0.03(0.88)	0.03(0.86)	0.16(0.45)	−0.12(0.57)
	APOA-II	−0.22(0.30)	−0.07(0.75)	−0.57(0.005)^a^	0.16(0.46)
	APOA-II/APOA-I ratio	−0.21(0.33)	−0.10(0.65)	−0.57(0.005)^a^	0.18(0.40)

*APOA-I* Apolipoprotein A-I, *APOA-II* Apolipoprotein A-II, *HDL* High Density Lipoprotein, *LCAT* Lecithin: cholesterol acyltransferase

^a^Correlation is significant at the *P*_*value*_ ≤0/05 level (2-tailed)

## Discussion

To the best of our knowledge, this study is the first attempt to investigate the effects of weight loss in -256 T>C polymorphism and to gain a better understanding of the differences between genotype in response to the same modification. In the present study, HDL concentration decreased significantly in both groups after intervention; however, the comparison of HDL subfractions showed that HDL2 decreased significantly in the TT/TC group, while HDL3 decreased significantly in the CC genotype group.

Weight loss is an important regulator of dyslipidemia [24]. When analyzing the effect of weight loss by calorie restriction on the HDL level, a meta-analysis of 70 studies indicated that, during active weight loss, the level of HDL reduces temporarily, but during the weight maintenance phase the level of HDL in serum increases. This study estimated that, with every kilogram of body weight loss, the concentration of HDL decreases by 0.27 mg/dl, but when the subject’s weight is stabilized, it increases by 0.35 mg/dl per kilogram of weight loss [25]. The responsible mechanism for reduced HDL during calorie restriction may consist of an increased degradation and/or decreased production of HDL particles, as well as calorie restriction, causing a decrease in the production of chylomicron-derived HDL particles [26].

The result of the Genetics of Coronary Artery Diseases in Alaska Natives (GOCADAN) study indicated that individual HDL subfractions may be influenced by different genetic variations [27]. As mentioned earlier, the replacement of T with C at the position −256 of APOA-II resulted in a decreased expression of APOA-II in the liver, while different genotypes of APOA-II have different serum APOA-II levels [13]. The effects of APOA-II on HDL size for the first time were observed in studies of inbreed strains of mice. In these studies, as the level of APOA-II increased, the size of HDL increased continuously [28, 29]. In addition, the studies of APOA-II transgenic mice confirmed the positive relation between higher levels of APOA-II and increased HDL size [30, 31].

Although these studies used other methods such as Electrophoresis [30] and Gel-filtration chromatography [31], their results are in agreement with the present study. We observed that, after weight loss, HDL3 was reduced significantly in the CC genotype group; on the other hand, the reduction of HDL2 in the CC genotype group (*P* = 0.95) in comparison with the TT/TC genotype group (*P* = 0.005) was much less. This shows that, after weight loss, the CC genotype shifts toward larger-sized HDL (Table 2). The ratio of APOA-II to APOA-I reduced significantly in the TT/TC group, while it did not change in the CC genotype (Table 2). Also, a negative correlation between APOA-II, ratio of APOA-II/APOA-I and HDL3 was seen in the CC genotype group (Table 4). It seems that APOA-II effects the HDL size by reducing the HDL ability to act as a substrate for HL [32]. In addition, the ratio of APOA-II to APOA-I is one of the important parameters of HDL function. Hedrich et al. claimed that, when the ratio of APOA-II to APOA-I increases, the HDL size becomes larger due to the inhibition of HL [32]. They have proposed that, in HDL with higher ratios of APOA-II to APOA-I, hydrolysis of phospholipid triglyceride by HL is reduced [32]. In addition, LCAT esterified cholesterol on the surface of HDL3 and the cholesterol esters are more hydrophobic than free cholesterol and they immigrate into the core of the lipoprotein, with the resulting of generation HDL2 particles in the plasma [8]. In the present study, LCAT activity increased after the intervention, but this was not significant. The results of other studies demonstrated increased LCAT activity after weight loss in dogs [33], and decreased significantly LCAT mass and activity at one month after weight loss by gastric bypass surgery in obese women [34]. The difference between the results might be due to different methods for measuring LCAT activity, where they used radioactivity [33] or measuring increased cholesterol ester in plasma as a result of LCAT activity [34]. As is shown in Table 4, there is a nonsignificant negative correlation between APOA-II and LCAT activity. APOA-I is an activator of LCAT, whereas APOA-II neither activates nor inhibits LCAT activity [35]. In addition, APOA-II is more hydrophilic than APOA-I and can inhibit LCAT activity indirectly by displacing APOA-I from the surface of HDL, or by hindering LCAT binding to APOA-I on HDL and inhibit cholesterol esterification [36]. However, to justify the result of HDL subfractions in this study, there is a need to measure other enzymes related to HDL metabolism. It has been proposed in other studies that APOA-II can alter other enzymes related to HDL, including the inhibition of cholesteryl ester transfer protein (CETP), phospholipid transfer protein (PLTP) and Endothelia lipase (EL) [37], which can have an influence on the maturation process of HDL subclasses. However, it was not possible for us to measure these enzymes.

The evidences regarding the pro-atherogenic or anti-atherogenic effect of HDL subfractions in humans are conflicting and confusing, and it is not clear whether it is related to HDL size or apolipoprotein composition, density, mobility or a combination of their properties [38]. In this regard, some studies indicate that smaller, dense HDLs are associated positively with CVD [39]. In contrast, Kontush et al. demonstrated that small, dense HDL has a potential role in protecting LDL from oxidation [40]. In another study, it was demonstrated that both HDL2 and HDL3 protect against CVD [41]. However, most studies that investigate the therapeutic modulation of HDL subfraction demonstrate that larger HDL particles are more protective against CVD [42]. In sum, according to a review article by Rizzo et al., further clinical investigations are required to clarify the physicochemical and functional heterogeneity of HDL subfractions [38].

## Conclusion

A comparison of mean changes of variables within two genotype groups showed that weight loss resulted from a reduction of HDL in both groups. However, in C homozygote carriers, it was shown that HDL3 reduced significantly and leads to a general shift toward larger size HDL subfractions after intervention, while in T allele carriers HDL2 decreased significantly and weight loss leads to shift toward smaller size HDL subfractions.

### Study limitation

The present study has some limitations. First, randomization was not carried out when selecting the patients and placing them in the two genotype groups, which might have had an influence the results. Second, due to the limitation in patients in the CC genotype group and exclusion criteria for selecting patients, there was no control group in each genotype. Third, due to financial limitations, we did not measure other enzymes related to HDL size such as CETP, HL, EL and PLTP. Finally, we have examined a short-term weight loss during a six-week period; therefore, a more prolonged course of weight maintenance could show the full benefits of weight loss and corroborate the findings.

## Abbreviations

APOA-I: Apolipoprotein A-I
APOA-II: Apolipoprotein A-II
AT: Activity Thermogenesis
BMI: Body Mass Index
CETP: Cholesteryl Ester Transfer Protein
EL: Endothelia Lipase
HDL-C: High Density Lipoprotein-Cholesterol
HL: Hepatic Lipase
LCAT: Lecithin: cholesterol acyltransferase
PLTP: Phospholipid Transfer Protein
RCT: Reverse Cholesterol Transfer
T2DM: Type 2 Diabetes Mellitus
TEE: Total Energy Expenditure
TEF: Thermic Effect of Food
